# Epigenome-wide skeletal muscle DNA methylation profiles at the background of distinct metabolic types and ryanodine receptor variation in pigs

**DOI:** 10.1186/s12864-019-5880-1

**Published:** 2019-06-13

**Authors:** Siriluck Ponsuksili, Nares Trakooljul, Sajjanar Basavaraj, Frieder Hadlich, Eduard Murani, Klaus Wimmers

**Affiliations:** 1 Leibniz Institute for Farm Animal Biology (FBN), Institute for Genome Biology, Functional Genome Analysis Research Unit, Wilhelm-Stahl-Allee 2, 18196 Dummerstorf, Rostock, Germany; 2 Faculty of Agricultural and Environmental Sciences, University Rostock, 18059 Rostock, Germany

**Keywords:** DNA methylation, Pig, RRBS, Genetic variation, RYR

## Abstract

**Background:**

Epigenetic variation may result from selection for complex traits related to metabolic processes or appear in the course of adaptation to mediate responses to exogenous stressors. Moreover epigenetic marks, in particular the DNA methylation state, of specific loci are driven by genetic variation. In this sense, polymorphism with major gene effects on metabolic and cell signaling processes, like the variation of the ryanodine receptors in skeletal muscle, may affect DNA methylation.

**Methods:**

DNA-Methylation profiles were generated applying Reduced Representation Bisulfite Sequencing (RRBS) on 17 Musculus longissimus dorsi samples. We examined DNA methylation in skeletal muscle of pig breeds differing in metabolic type, Duroc and Pietrain. We also included F2 crosses of these breeds to get a first clue to DNA methylation sites that may contribute to breed differences. Moreover, we compared DNA methylation in muscle tissue of Pietrain pigs differing in genotypes at the gene encoding the Ca2+ release channel (RYR1) that largely affects muscle physiology.

**Results:**

More than 2000 differently methylated sites were found between breeds including changes in methylation profiles of *METRNL, IDH3B, COMMD6*, and *SLC22A18*, genes involved in lipid metabolism. Depending on RYR1 genotype there were 1060 differently methylated sites including some functionally related genes, such as *CABP2* and *EHD,* which play a role in buffering free cytosolic Ca^2+^ or interact with the Na^+^/Ca^2+^ exchanger.

**Conclusions:**

The change in the level of methylation between the breeds is probably the result of the long-term selection process for quantitative traits involving an infinite number of genes, or it may be the result of a major gene mutation that plays an important role in muscle metabolism and triggers extensive compensatory processes.

**Electronic supplementary material:**

The online version of this article (10.1186/s12864-019-5880-1) contains supplementary material, which is available to authorized users.

## Background

Epigenetic modifications of the genome can have short-term and long-term influence on gene expression under different environment [1]. In turn, these changes in expression profiles have implications for traits associated with physical and metabolic integrity [2]. Epigenetic regulation of gene activity is based on chemical modifications of DNA and chromatin, such as DNA methylation or histone acetylation, methylation, phosphorylation, and ubiquitination. Variation in DNA methylation relate to a wide range of cellular functions and pathologies, and the role of DNA methylation dynamics on skeletal muscle development and disease have been recently described [3].

Regulation of DNA methylation and demethylation during cellular differentiation and tissue specification is more dynamic than previously thought [3]. Most genome-wide DNA methylation changes in skeletal muscle have been analysed based on aging in pigs and humans, and the results highlight the role of DNA methylation changes in enhancing proteolysis, a protein catabolic process that is relevant to muscle tissue function and neuromuscular junctions [4, 5]. These studies emphasize the importance of epigenetic mechanisms in age-related muscle disease.

In addition, differences in DNA methylation contribute to phenotype even in monozygotic twins or cloned animals [6–8]. Further, DNA methylation not only influences individual genetic variation, but also reaches population-level differences. Methylome variation has been demonstrated in Caucasian, Asian, and African humans with population-specific DNA methylation sites, along with heritability of variation in DNA methylation [9]. In addition, genetic variants at or near CpG sites change gene expression and can modulate methylation status. This has been attributed to variability in DNA methylation that can be explained by genetic variation at the CpG site itself [10].

Pigs are an important food source and human medical research model [11]. Long term selection and breeding of pigs has resulted in both genetic variation and epigenetic modification [12–15]. Genome-wide DNA methylation profiling in adipose and skeletal muscle tissues of three pig breeds reveals methylation of the promoter regions of genes linked to fatness [16]. Duroc and Pietrain are two common commercial pig breeds known for their divergence in growth rate, body composition, muscularity, and fat content; Pietrain is leaner and Duroc is more obese. Epigenetic variation may have contributed to the selection progress. Epigenetic variation may serve as an adaptation mechanism mediating the response to exogenous stressors. Both processes may have led to the evolution of new alleles.

We sought to catalogue DNA methylation sites at single-base resolution in the muscles of several pig breeds with distinct metabolic types using reduced-representation bisulfite sequencing (RRBS). RRBS is a cost-effective method that allows scalable genome-wide analysis of DNA methylomes with single-base resolution [17]. We analysed DNA methylation variation in distinct metabolic types of pig breeds: Duroc, Pietrain, and a F2 cross between the two breeds. Moreover, within the Pietrain breed, we considered animals of both homozygous genotypes at the *RYR1* g.1843C > T SNP that causes malignant hyperthermia syndrome (MHS) in pigs. We identified genome-wide DNA methylation patterns related to metabolic distinctness due to long-term selection (Duroc and Pietrain breeds), F2 generation cross-breeding between Duroc and Pietrain breeds (DuPi), and differences at a major gene within a pig breed (PiPP and PiNN). This study shows that DNA methylation differs in numerous genes due to long-term selection between breeds as well as due to the large impact of a single major gene.

## Methods

### DNA isolation and library construction

Duroc, Pietrain (MHS homozygous negative (PiNN) and positive (PiPP), respectively) and the F2-Duroc-Pietrain-Ressource Population of the University of Bonn (MHS homozygote negative, DuPi), were fattened at the “Lehr- und Forschungsstation Frankenforst” – a department of the University of Bonn. Pure-bred animals of the breeds Pietrain and Duroc originate from the same commercial breeding lines, which were used to build-up the F2-population. PiPP and PiNN belonged to a line segregating at the porcine ryanodine receptor 1 gene mutation (*RYR1*, C1843T). Animals of the breed Pietrain were genotyped at RYR1, C1843T to identify homozygous MHS negative founders of the DuPi populations and members of the two groups, PiNN and PiPP. Pigs samples in this study were sub-grouped based on our previous study, in which phenotype details have been reported [18, 19]. During the fattening period all pigs received a diet consisting of 13.4 MJ ME/kg, 16% crude protein, 0.75% calcium and 0.55% phosphorus. After slaughter, tissue samples from the longissimus muscle between the 13th and 14th thoracic vertebrae (Duroc, *n* = 5; DuPi, n = 5; PiNN *n* = 3, *RYR1* g.1843C/C; PiPP, n = 5, *RYR1* g.1843 T/T) were collected from each breed for DNA isolation. Phenotypes, gender and age of the individual samples are shown in Additional file 1. Muscle samples were frozen in liquid nitrogen and stored at − 80 °C until analysis.

RRBS is a method designed to integrate restriction enzyme digestion, bisulfite conversion, and next-generation sequencing (NGS) to analyse methylation patterns [20]. RRBS with double enzyme (*Msp*I and *Taq*I) digestion and increased selected-fragment size was used to enhance genome-wide CpG coverage. To construct the RRBS library, 2 μg of DNA with a 1% spike-in control (unmethylated cl857 Sam7 Lambda DNA, Promega) was digested with *Msp*I and *Taq*αI. Multiplexing of several samples per sequencing lane with the Illumina TruSeq DNA library preparation kit was used. Purified digested DNA fragments were end-repaired, A-tailed, and ligated to C-methylated adapters using a TruSeq Nano DNA Sample Preparation kit (Illumina) according to the manufacturer’s recommendations. Next, adapter-ligated DNA fragments were size-selected on 2% low-range ultra-agarose gels to obtain inserts of 40–240 bp. The purified DNA library was subjected to bisulfite conversion using an EpiTect Bisulfite kit (Qiagen). PCR amplification (95 °C for 3 min, followed by 10 cycles of 98 °C for 20 s, 60 °C for 15 s and 72C for 30 s) of the library was performed using a PfuTurbo Cx Hotstart DNA Polymerase kit (Stratagene). The quality of RRBS libraries was assessed using an Agilent DNA 1000 kit (Agilent Technologies). NGS of RRBS libraries was performed on an Illumina HiSeq2500 for single-reads of 114 bp at the FBN Dummerstorf. The bcl2fastq2 conversion software v2.19 was used to convert base call files from a sequencing run into FASTQ files. The sequence reads were mapped to the pre-converted reference genome (Sscrofa 11.1), reads aligned to the multiple regions were removed, and best uniquely mapped reads were used for methylation calling. In total, 17 RRBS libraries passed quality control and were used for further analysis.

### RRBS data analysis

A standard analysis pipeline of DNA methylation involving pre-processing and read-alignment per CpG methylation call and identification of differentially methylated CpG sites/regions has been established by our group. The raw fastq files were pre-processed using a custom C++ based program to retain sequence reads with a mean Phred quality (Q-score) > 20, a minimum length of 30 bp without uncertain base-calling of N, and adapter sequence contamination. Two bases of both 5′- and 3′- fragment ends, which were artificially filled-in to create blunt-ends and to facilitate adapter-ligation during library construction, were removed. Clean reads were further passed to the read-alignment step using a default setting of Bismark version 0.19.0 [21], which maps bisulfite sequencing reads to the reference genome (Sscrofa 11.1) using the short read aligner Bowtie2 version 2.2.8 and further performs methylation calls for each cytosine in CpG, CHG, and CHH contexts (where H is A, C, or T).

The final differential methylation analysis was done using the R-based software tool methylKit version 1.8.0 [22]. CpGs sites covering less than 10X were removed based on methylKit’s proposed quality control [22]. In addition, the reads showing no methylation variation across all samples were filtered out. Logistic regression of the MethylKit was applied to evaluate methylation proportion of each CpG between samples [22].

The standard false discovery rate (FDR)-based method was used for multiple hypothesis testing [23]. Top differentially methylated CpG sites from each pairwise comparison were selected based on FDR values (FDR < 0.05). Moreover, we considered only CpGs with > 25% differences in DNA methylation levels and focused on CpG sites in within 2 kb of the transcription start site (TSS) to prioritize variable sites for consideration in future analyses. Differentially methylated CpG sites were annotated to genomic features using the genomation R/Bioconductor package (version 1.16.0). In order to identify methylated sites that are of potential origin of either the Pietrain or the Duroc breed, comparisons between DuPi on the one hand and the pure breeds on the other hand were made revealing those methylation sites that were different to one of the pure breeds only, but not to the other pure breed. Accordingly, ‘Duroc-origin’ means that there is no significant difference between DuPi and Duroc, but there is differential methylation of DuPi vs. PiNN or PiPP; ‘Pietrain-origin’ means that there is no differential methylation of DuPi vs. PiPP or PiNN or both, but there is differential methylation of DuPi vs. Duroc.

### SNP discovery

The non-bisulfite-treated version of reduced representation DNA libraries of a pool of each breed (4 libraries) were sequenced for SNP identification. Raw reads from non-bisulfite treatment were pre-processed similar to those from bisulfite conversion, i.e. a mean Q-Score of > 20, min. Length of > 30 bp, no N base-calls, no adapter sequence, and a 2 bp-trimming from both fragment ends. Quality-filtered reads were then aligned to the porcine reference genome Sscrofa 11.1 using Bowtie 2 version 2.2.8 [24]. Uniquely aligned reads and dbSNP build 150 (ftp://ftp.ncbi.nih.gov/snp/organisms/archive/pig_9823/) were used for variant identification using GATK version 3.7 with default parameters [25]. These variant sites were removed from the RRBS dataset before analysis.

### Bisulfite PCR and pyrosequencing

Differentially methylated CpGs identified by genome-wide analysis were validated in 10 animals per breed including the ones used for RRBS analysis by bisulfite PCR and pyrosequencing methods. Genomic DNA from skeletal muscle tissue was treated with bisulfite using the EZ DNA Methylation Gold Kit (Zymo Research) according to supplier’s instructions. Primers were designed using pyrosequencing assay design software and listed in Additional file 2. Converted DNA was amplified by PCR using AmpliTaq Gold DNA polymerase (Applied Biosystems, Cat. No. 4311814): hot start at 94 °C for 4 min; 40 cycles of 94 °C for 30 s, primer-specific annealing temperature for 40 s, and 72 °C for 1 min; and 72 °C for 8 min. Pyrosequencing of PCR products was performed using PSQ™96MA per manufacturer’s instructions (Qiagen). CpG methylation percentages were calculated using PSQ96MA System software 2.02 based on the height of T and C peaks at target CpG sites.

### Expression pattern of Duroc and PiNN

We previously analysed expression of genes in longissimus muscles from the same animals in Duroc and PiNN (*n* = 10 per breed) using Porcine Snowball Microarray (Affymetrix) [18]. The 5 Duroc and 3 PiNN animals that underwent RRBS analysis in this study were a subset of those of our previously expression study. Expression data are available in the Gene Expression Omnibus public repository with the GEO accession number GSE69840: GSM1709900–GSM1709919. Differential expression analysis was performed using ANOVA in JMP Genomics 7 (SAS Institute). Breed was treated as a fixed effect. To control for multiple testing, *p*-values were converted to a set of *q*-values [26].

### Functional analysis

Functional network analysis was done to gain biological insights into differentially methylated loci between pig breeds. Genes annotated from the selected CpG (different methylation level > 25%, significant at FDR < 5%, position < 2 kb from TSS) were included in the gene function network analysis and GO enrichment analysis. Ingenuity pathway analysis (IPA, Ingenuity Systems, Inc., CA, USA) with its core analysis features was used. IPA categorizes genes based on annotated gene functions and statistical tests for over-representation of functional terms within a gene list using Fisher’s Exact Test. The online tool DAVID version 6.8 was used to perform an enrichment analysis in GO-ontology terms.

### Quantitative real-time PCR (qPCR)

Total RNA was isolated by using Tri-Reagent-phenol-chloroform extraction (Sigma-Aldrich, Taufkirchen, Germany) according to manufacturer’s protocol. To remove any DNA DNase (Qiagen, Hilden, Germany) treatment and purification using the RNeasy Mini Kit (Qiagen, Hilden, Germany) were performed. To check, whether the RNA samples still contain DNA, PCR was performed on RNA samples without cDNA synthesis using primers for the glycerol aldehyde-3-phosphate dehydrogenase (*GAPDH*) gene. Muscle cDNA was synthesized from samples of the identical 10 animals per breed that were used to validate the differentially methylated CpG sites. QPCR was performed using the LightCycler 480 Real-Time PCR System (Roche Diagnostics). Amplification was conducted in duplicate according to the supplier’s instructions. Reactions were performed in a final volume of 10 μL using 5.0 μL of LightCycler 480 SYBR Green I Master (Roche), 2.0 μL of Aqua Dest water, 10 μM of each primer, and 40 ng of cDNA. Temperature profiles comprised an initial denaturation step at 95 °C for 10 min and 40 cycles of denaturation at 95 °C for 15 s, annealing at 60 °C for 10 s, and extension at 72 °C for 15 s. Primer sequences are provided in Additional file 2. Expression levels were normalised to *RPL32, RPS11,* and *ß-ACTB*.

## Results

### Genome wide DNA methylation profiling of muscle

**Fig. 1:**
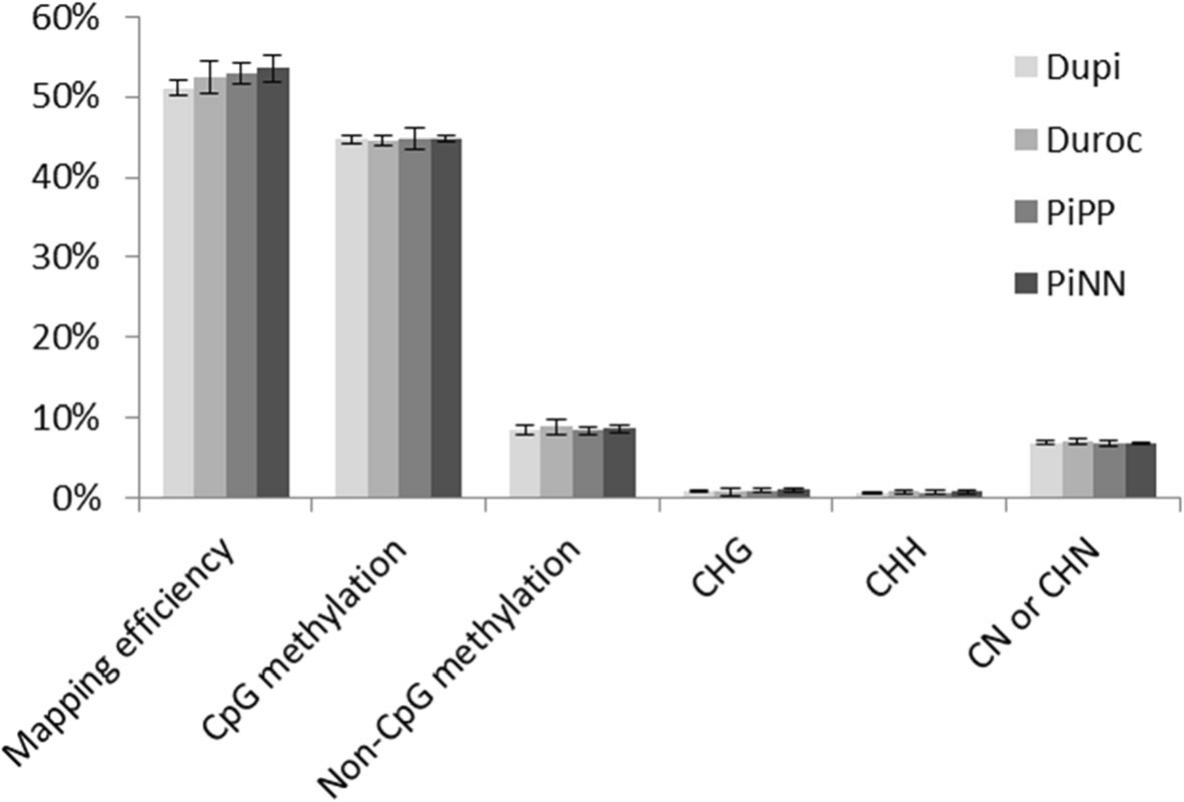
Comparison of the mapping efficiency and methylation level of CpG and non-CpG sites between 4 pig breeds. Non-CpG methylation was divided into CHG, CHH, CN, or CHN

**Fig. 2:**
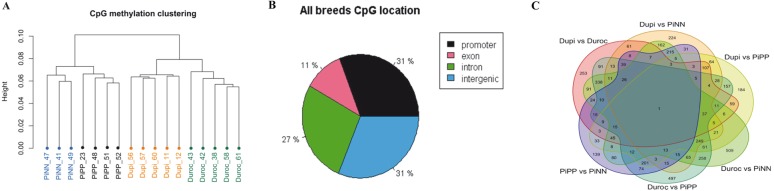
DNA methylation profiling of muscle in divergent pig breeds with distinct metabolic types and genetic backgrounds. **a** Hierarchial cluster analysis of individual samples of all 441,894 CpGs from 4 pig breeds with at least 10-fold coverage. Distance of the sample according to methylation patterns was estimated by ward method using methykit R packages. **b** Mapping location of all CpGs on promoter, exon, intron, and intergenic regions are given as percentages. **c** Venn diagram of the number of differentially methylated CpGs between breeds

We sequenced 18 RRBS libraries using a single-read flow cell with 114 cycles on a HiSeq2500. We used 17 RRBS libraries with an average of 30 million high-quality reads per library (Table 1). The average mapping efficiency was 52.4 ± 1.6% using Bismark run with Bowtie 2 against a reference pig genome (Sscrofa11.1). Overall methylated cytosines in the CG/CHG/CHH (whereby H can be either A, T or C) context were 44.7%/0.9%/0.7% in DuPi, 44.5%/0.8%/0.80% in Duroc, 44.2%/1.0%/0.8% in PiPP, and 44.8%/1%/0.8% in PiNN, respectively, with a bisulfite conversion rate of > 99.0%. C methylated in unknown context like CN or CHN (whereby N can be either A, T, G or C) was observed to be 6.98% in DuPi, 7.14% in Duroc, 6.65% in PiPP and 6.90% in PiNN. Figure 1 shows mapping efficiencies of CpG- and non-CpG-methylation sites (CHG, CHH, CN, or CHN) in muscle tissue of 4 pig populations. In total, 441,894 CpG positions were identified for further study after quality checks and normalization with at least 10X coverage. The hierarchical clustering dendrogram of all samples revealed a specific, distinct methylation pattern in each breed (Fig. 2a).

**Table 1:** Details of mapping RRBS libraries to the porcine genome (Sscrofa11.1) using bismark (Bowtie 2)

Sample ID	Clean reads	Unique alignments	Mapping efficiency	CpG methylation	Non-CpG methylation
DuPi_11	25,598,295	13,016,706	50.8%	44.0%	8.1%
DuPi_12	33,475,531	16,674,560	49.8%	45.2%	8.7%
DuPi_56	33,731,881	17,385,378	51.5%	45.2%	8.0%
DuPi_57	29,270,325	15,359,097	52.5%	44.8%	8.5%
DuPi_60	31,094,984	15,763,028	50.7%	44.2%	9.4%
Duroc_38	28,869,589	14,725,935	51.0%	43.7%	8.1%
Duroc_42	23,517,510	12,022,839	51.1%	44.8%	9.1%
Duroc_43	35,098,019	19,596,737	55.8%	45.2%	10.2%
Duroc_58	35,688,527	18,941,344	53.1%	44.5%	9.4%
Duroc_61	30,005,918	15,453,489	51.5%	44.5%	8.0%
PiPP_23	30,088,721	15,743,823	52.3%	44.7%	9.3%
PiPP_48	28,917,500	15,104,512	52.2%	43.3%	8.2%
PiPP_51	34,649,573	18,386,095	53.1%	44.6%	8.6%
PiPP_52	29,679,615	15,433,031	52.0%	44.1%	7.9%
PiNN_41	31,118,398	16,512,344	53.1%	44.5%	9.0%
PiNN_47	25,844,090	13,465,314	52.1%	44.6%	8.2%
PiNN_49	30,608,401	16,929,760	55.3%	45.3%	9.0%

By screening SNPs from 441,894 CpG positions using our own sequence data, we found about ~ 1.1% (4849/441894) SNPs at the CpG sites. This 1.1% of polymorphic sites at CpG positions was excluded from further analysis. In addition, CpG sites with 0% or 100% methylation in all samples were deleted. After applying these filters, 437,045 CpGs were used for differential CpG methylation analysis among breeds. Among the remaining 437,045 CpGs, 31% were located on promoter regions, 11% on exons, 27% on introns, and 31% on intergenic regions (Fig. 2b).

#### Identification of differentially methylated CpGs between breeds

In total, 4626 CpG positions were differentially methylated between any of the 4 groups of pigs at FDR < 5% with a > 25% methylation difference. A summary of differentially methylated CpGs between breeds in functional regions of the genome is shown in Table 2. The most differentially methylated CpGs were found between Duroc and PiNN (2303 CpGs), followed by Duroc and PiPP (2276 CpGs). 1060 CpGs were differentially methylated between PiPP and PiNN. Our analysis showed 509, 497, 253, 224, 184, and 139 CpGs with specific differential methylation between Duroc vs PiNN, Duroc vs PiPP, DuPi vs Duroc, DuPi vs PiNN, DuPi vs PiPP, and PiPP vs PiNN, respectively (Fig. 2c).

**Table 2:** Differentially methylated CpGs and their location in genomes of different pig breeds with FDR of < 5% and methylation difference of > 25%

Breed	No. of differentially methylation CpGs	Hypermethylation breeds (No.)	Location of differentially methylated CpGs		
			Exons	Introns	Promoter region around TSS (within 2 kb)
DuPi vs Duroc	1303	Duroc (846)	55	252	60
DuPi vs PiPP	1352	PiPP (601)	79	268	73
DuPi vs PiNN	1440	PiNN (650)	62	281	61
Duroc vs PiPP	2276	PiPP (861)	110	432	108
Duroc vs PiNN	2303	PiNN (899)	102	438	119
PiPP vs PiNN	1060	PiNN (549)	33	222	55

#### Comparison of CpG methylation levels between DuPi and Duroc

**Fig. 3:**
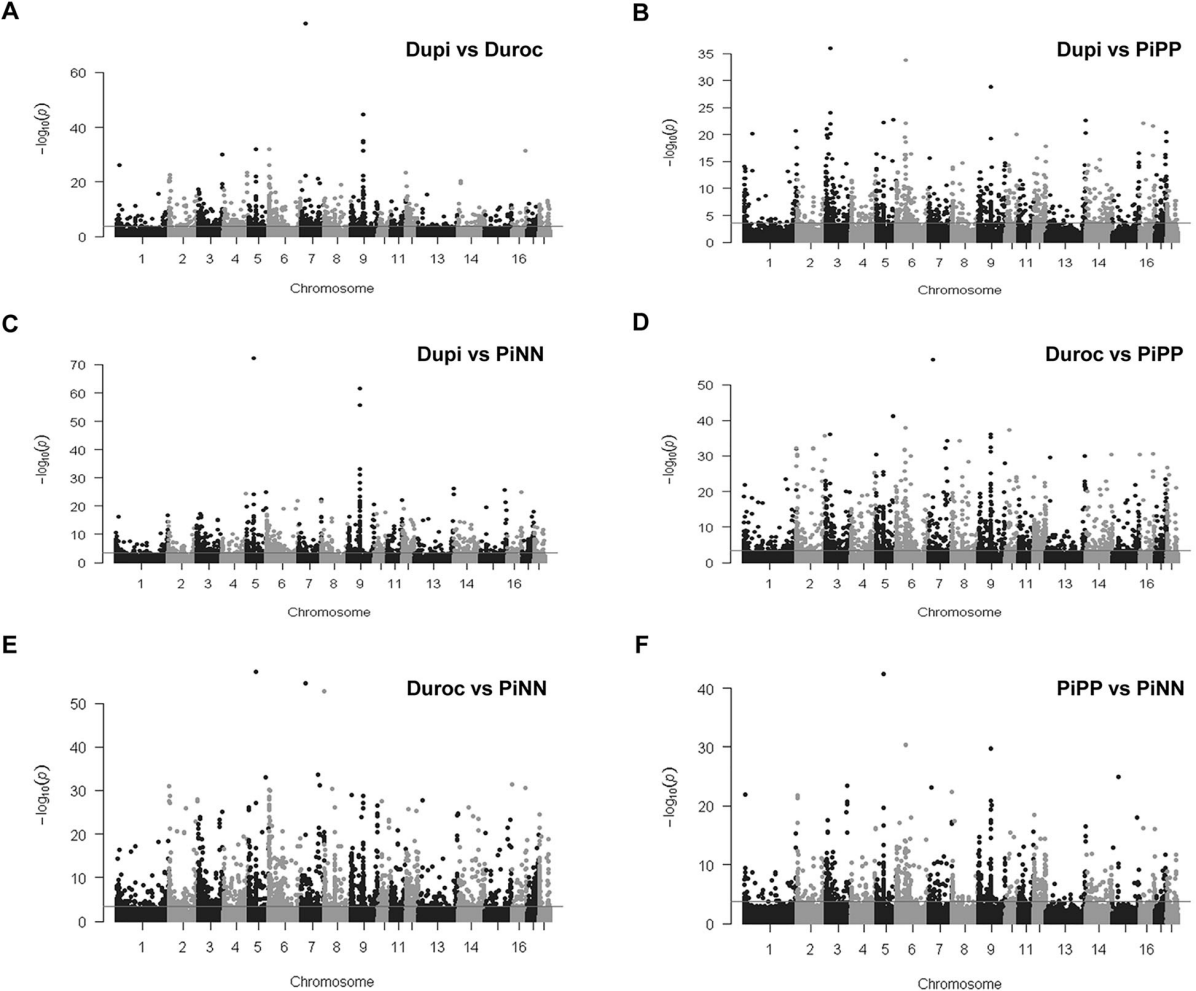
Manhattan plots showing the distribution of differentially methylated CpGs sites between breeds identified across all pig chromosome regions. **a** Differential methylation analysis between DuPi and Duroc. **b** Differential methylation analysis between DuPi and PiPP. **c** Differential methylation analysis between DuPi and PiNN. **d** Differential methylation analysis between Duroc and PiPP. **e** Differential methylation analysis between Duroc and PiNN. **f** Differential methylation analysis between PiPP and PiNN. Each point represents a CpG site, with genomic position on the x-axis and –log10 of the *p*-value for differential methylation between breeds on the y-axis. Red line represents significance at FDR < 5%. Chromosomes are alternately black and grey for ease of visibility

**Fig. 4:**
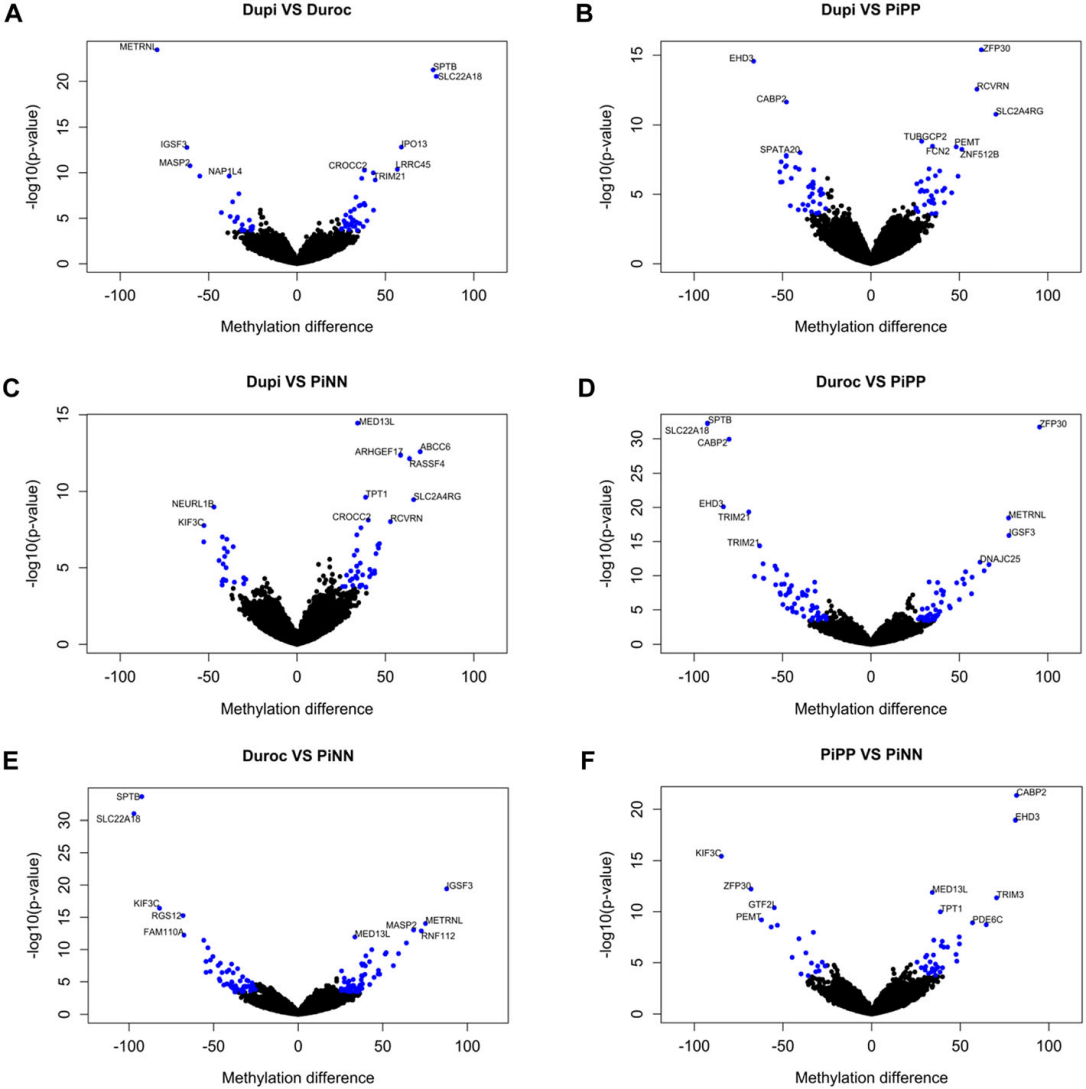
Volcano plots of differentially methylated CpGs in pairwise comparisons between breeds. **a** Differential methylated CpGs DuPi vs. Duroc. **b** Differential methylated CpGs DuPi vs. PiPP. **c** Differential methylated CpGs DuPi vs. PiNN. **d** Differential methylated CpGs Duroc vs. PiPP. **e** Differential methylated CpGs Duroc vs. PiNN. **f** Differential methylated CpGs PiPP vs. PiNN. The x-axis indicates differences in mean methylation percentages and the y-axis indicates negative log (*p*-values). Blue CpG sites were significant at FDR < 5%, showed methylation differences more or less than 25% and were located within 2 kb distance from TSS. Top ten genes annotated in the defined regions are shown

Manhattan plots were generated to show the distribution of differentially methylated CpGs sites among all 437,045 CpGs across all autosomal regions (Fig. 3). Differential methylation analysis revealed a total of 1303 CpGs sites were differentially methylated between DuPi and Duroc (Additional file 3). In total 55, 252, and 60 out of 1303 CpGs were located in exons, introns, or promoters, respectively (Table 2). Of the 1303 CpG positions, 846 were more methylated in Duroc than DuPi. Figure 4a shows a volcano plot depicting only annotated CpGs located in promoter regions within 2 kb of TSS. Most interestingly, the CpG site (SSC2, position 2,033,932 bp) was located in the promoter of *SLC22A18.* This CpG position was hypermethylated in Duroc and hypomethylated in DuPi. Similar CpGs with higher methylation in Duroc were also found in *SPTB, IP013, LRRC45, CROCC2,* and *TRIM21* (Additional file 3, Fig. 4a). CpG positions hypermethylated in DuPi were found within the promoters of *METRNL, IGSF3*, *MASP2,* and *NAP1L4*.

#### Comparison of CpG methylation levels between DuPi and Pietrain (PiNN and PiPP)

Manhattan plots of Fig. 3b and c show the distribution of differentially methylated CpG sites between DuPi vs PiPP and DuPi vs PiNN located on pig chromosome regions. Differential methylation analysis revealed a total of 1352 and 1440 CpGs sites differentially methylated between DuPi vs PiPP and DuPi vs PiNN, respectively (Additional files 4 and 5). The most prominent differentially methylated CpG sites between DuPi and PiPP were found within 22 Mb on SSC3 and within 47 Mb on SSC6. In total, 79, 268, and 73 differentially methylated CpG sites between DuPi and PiPP and 62, 281, and 61 differentially methylated CpG sites between DuPi and PiNN were found in exons, introns, and promoters, respectively (Table 2, Additional files 4 and 5).

Volcano plots of differentially methylated CpGs located in the promoters region around TSS within 2 kb are shown in Fig. 4b and c, highlighting the higher methylation levels of *EHD3*, *CABP2,* and *SPATA20* in DuPi and higher methylation levels of *ZEP30, RCVRN, SLC2A4RG, TUBGCP2, PEMT, FCN2,* and *ZNF512B* in PiPP (Additional file 4, Fig. 4b). Top differentially methylated CpG sites were located in promoters, with higher methylation levels in DuPi compared to PiNN, including *KIF3C* and *NEURL1B*. CpG sites more methylated in PiNN compared to DuPi included *MED13L, ABCC6, ARHGEF17, RASSF4, TP1, SLC2A4RG, CROCC2,* and *RCVRN* (Additional file 5, Fig. 4c).

#### Comparison of CpG methylation levels between Duroc and Pietrain (PiNN and PiPP)

Differential methylation analysis revealed a total of 2276 and 2303 CpG sites that were differentially methylated in Duroc vs PiPP and Duroc vs PiNN breeds, respectively (Additional files 6 and 7, Fig. 3d and e). The location of differentially methylated CpG sites between Duroc and Pietrain is summarized in Table 2. About 1268 CpGs were common in the comparisons between Duroc with PiPP and with PiNN. This includes CpGs at position 63.38–63.39 kb on SSC9, which had less methylation in Duroc than Pietrain. Many CpGs located in promoter regions, including *SPTB, SLC22A18,* and *TRIM21,* were highly methylated in Duroc and unmethylated in both PiPP and PiNN (Fig. 4d and e). CpGs in *METRNL, IGSF3,* and *RNF112* were highly methylated in PiPP or PiNN and unmethylated in Duroc (Fig. 4d and e).

#### Comparison of CpG methylation levels between Pietrain breeds

Figure 3f represents the distribution of differentially methylated CpG sites in PiPP vs PiNN along chromosome regions. Differential methylation analysis between Pietrain breeds revealed a total of 1060 CpG sites (Additional file 8). The most interesting and highly methylated CpGs located on SSC3 were within 112–113 kb, which includes CpGs on *EHD3*. In total 33, 222, and 55 differentially methylated CpGs between PiPP and PiNN were located in exons, introns, and promoter regions, respectively (Table 2, Additional file 8). CpGs located in promoters such as *CABP2, EHD3, MED13L, TRIM3, TPT1,* and *PDE6C* were more methylated in PiNN, whereas *KIF3C, ZFP30, GTF2L,* and *PEMT* were more methylated in PiPP (Fig. 4f).

#### Differences in expression and methylation levels between Duroc and PiNN

To evaluate the influence of DNA methylation on gene expression, we analysed our previous muscle expression profile from the same samples collected from Duroc and PiNN animals [18]. We selected genes that are present on the microarrays and are close to significant differentially methylated CpGs. The distance between this CpGs sites and the TSS of these genes is shown in column ‘dist_to_feature’ in Additional file 9. Two thousand three hundred three differentially methylated CpGs were found between Duroc and PiNN, of which 1128 were represented as probe sets on the microarrays. Out of 1128 probe sets, 269 were differentially expressed between Duroc and PiNN at *p* < 0.05, corresponding to *q* < 0.09. Considering a window of 10 kb around TSS, as in another study [27], there are 54 out of these 269 transcripts. Out of these 35 (65.8%) showed a negative correlation between expression and methylation (Additional file 9). These 9 out of 35 showed differentially methylated CpGs between Duroc and PiNN were located within 2 kb of the TSS of *ZNF740, MED13L, MPRIP, DEDD, IDH3B, COMMD6, HMGB2, IPO13,* and *ZNF24.*

#### Duroc- and Pietrain-origin in F2 DuPi

**Fig. 5:**
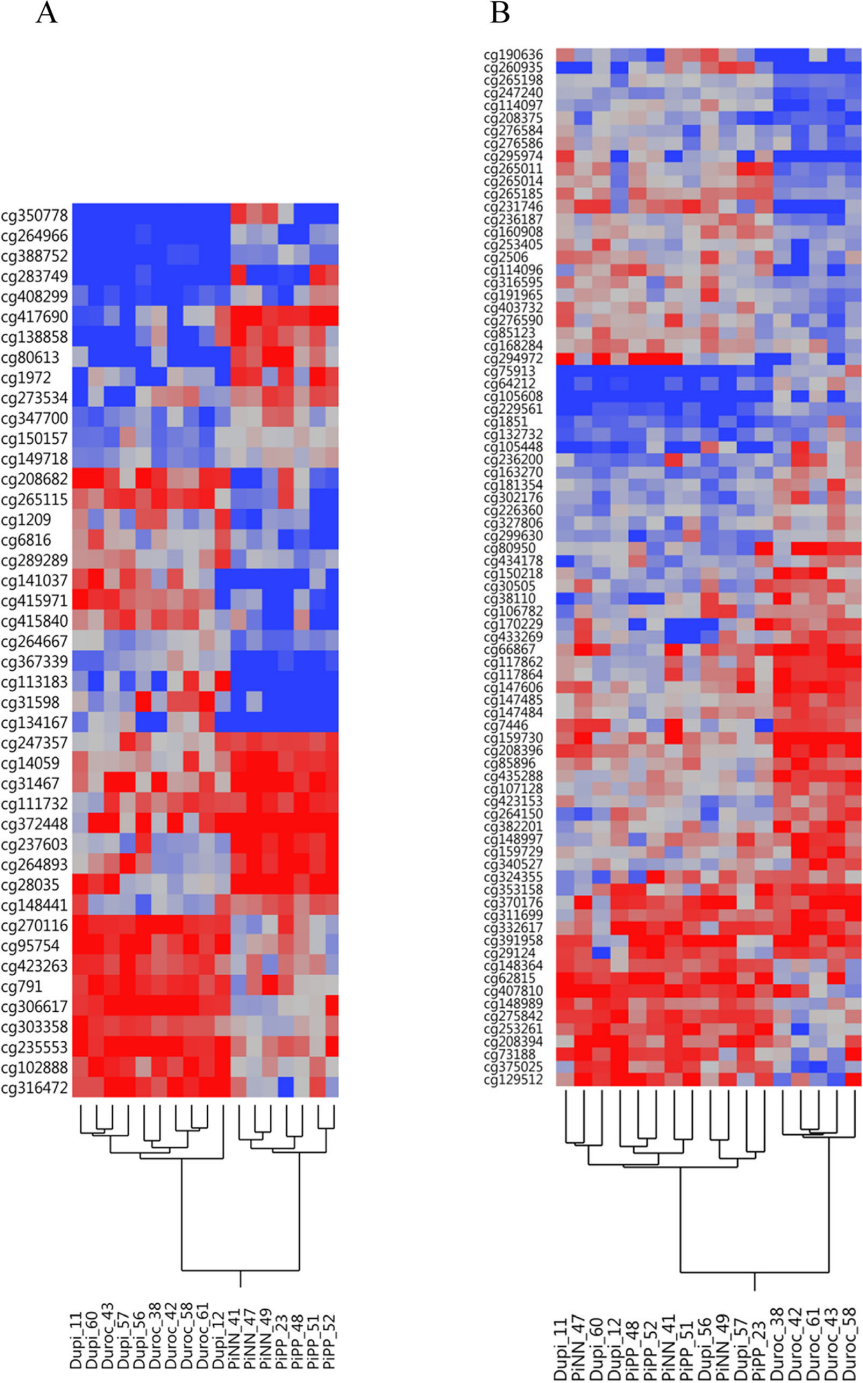
Heatmap and cluster analysis based on methylation levels of CpGs of Duroc-origin (**a**) and Pietrain-origin (**b**), respectively

Comparisons of the methylated sites in DuPi on the one hand and Duroc and PiNN/PiPP on the other hand revealed methylation sites of potential origin from either of the pure breeds. In fact, we identified 408 methylation sites that were differentially methylated between DuPi and either PiNN or PiPP or both, but not to Duroc, indicating potential Duroc-origin of these sites. The cluster analysis of this 408 CpGs sites is shown in Fig. 5a, where Duroc and DuPi cluster together. Similarly, we found 804 CpGs sites different between DuPi and Duroc, but not to PiNN or PiPP or both, indicating Pietrain-origin of these methylation sites. The cluster analysis of this 804 CpGs sites is shown in Fig. 5b, where Pietrain and DuPi cluster together.

#### Functional analysis of differentially methylated genes

**Fig. 6:**
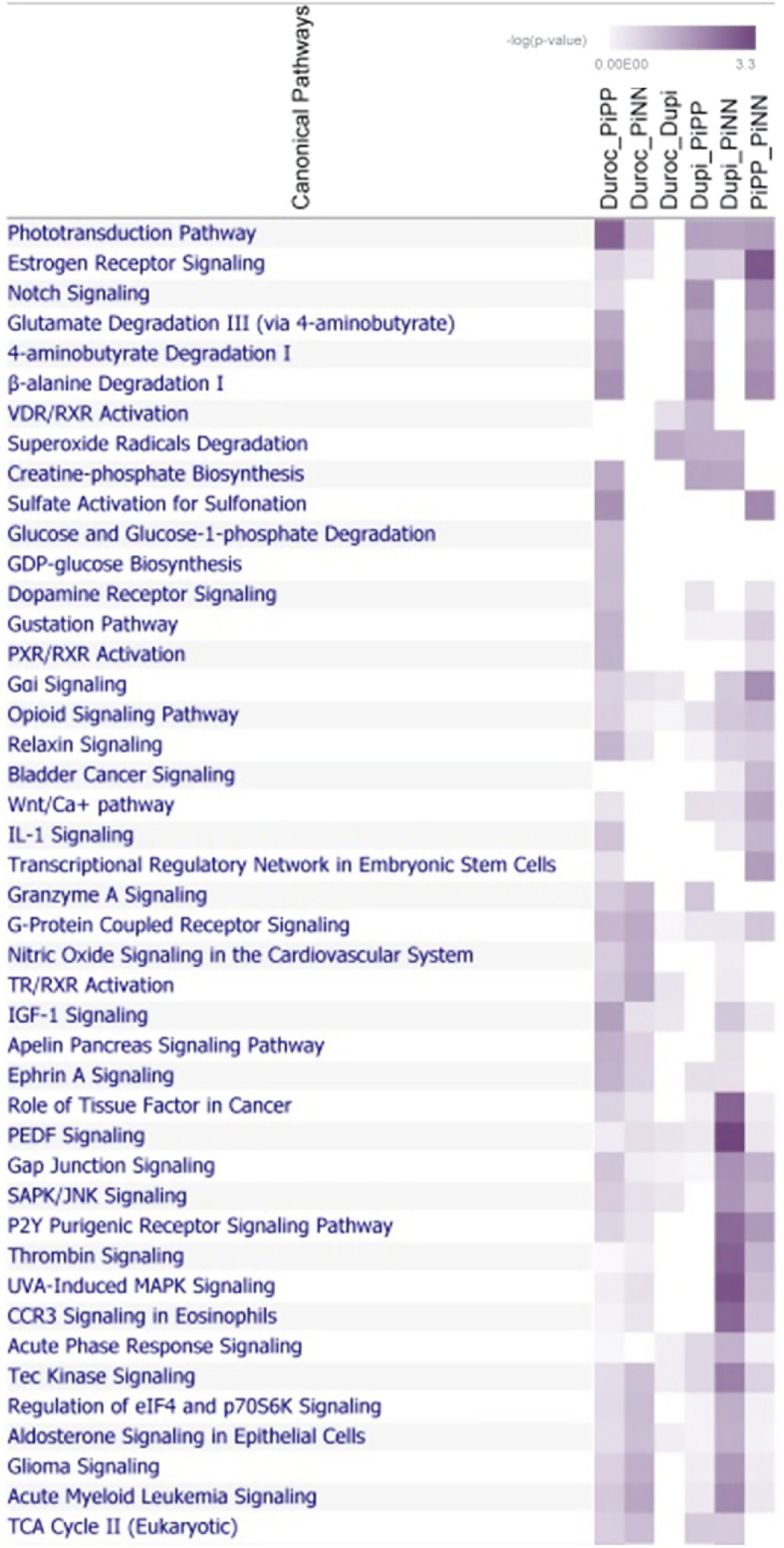
Heatmap indicating the enrichment of differently methylated transcripts between pig breeds in various canonical pathways; intensity of color indicates significance from light to dark

The annotated genes associated with differentially methylated CpG sites were subjected to a functional analysis. The significant canonical pathways (*p* < 0.05) are shown in Fig. 6, including some interesting molecular routes such as TR/RXR activation, which were found to be enriched only the comparisons of Duroc and other breeds. Other pathways, such as glucose and glucose-1-phosphate degradation and GDP-glucose biosysthesis, were found to be enriched in comparisons between Duroc and PiPP. The Wnt/Ca + pathway was found in the comparisons Duroc-PiPP, Dupi-PiPP, Dupi-PiNN or PiNN-PiPP. The GO enrichment analysis is shown in Additional file 10. Seven genes were found (*CABP2, OTOF, TPT1, DLL1, PCDHGC4, MMP28* and *EHD3*) enriched in GO:0005509~calcium ion binding when comparing between PiPP and PiNN.

#### Validation of differential methylation and expression profiles among breeds

**Fig. 7:**
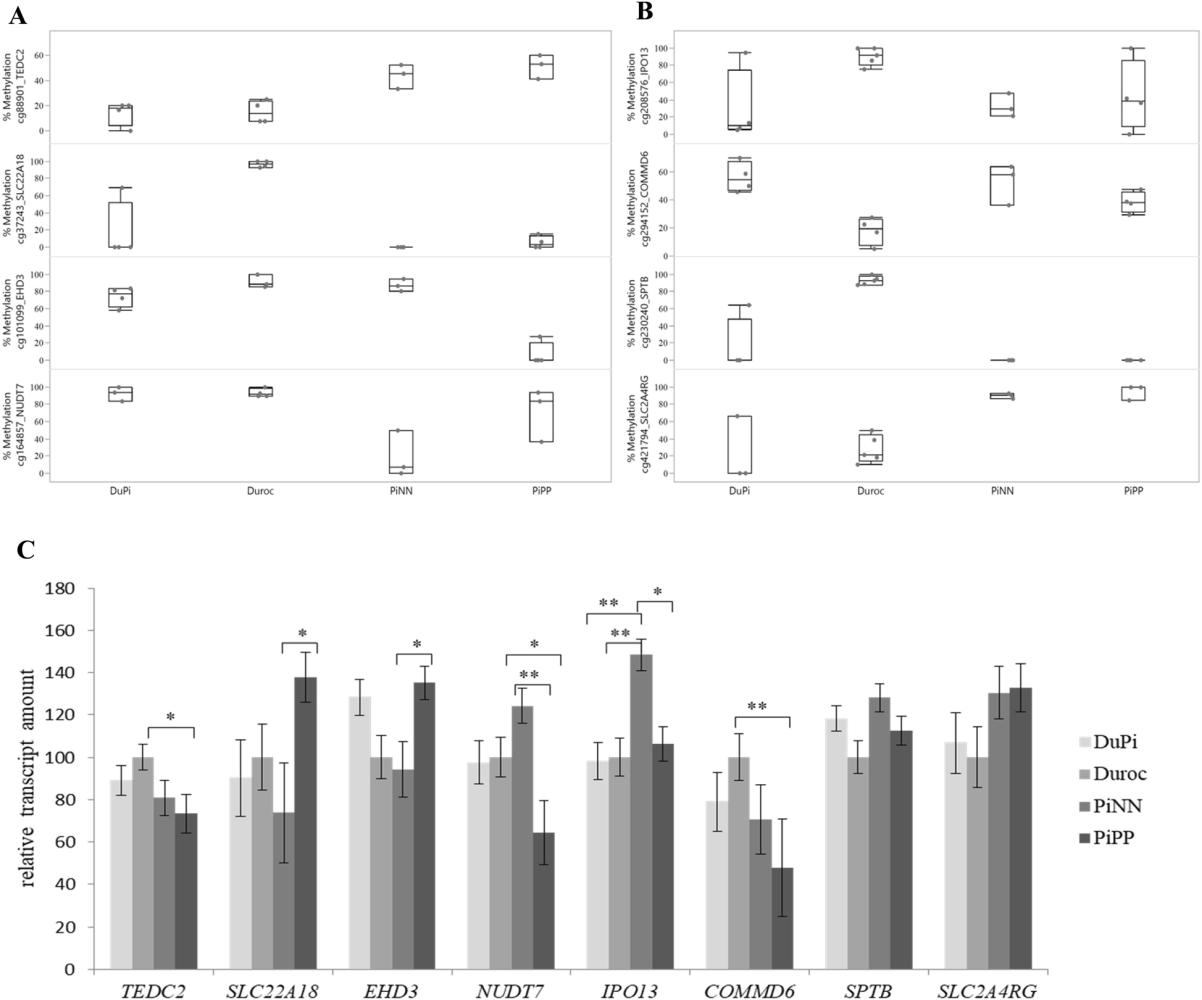
Differentially methylated CpG sites identified between pig breeds. **a** Box plot of percent methylation of CpG sites at *TEDC2, SLC22A18, EHD3,* and *NUDT7.* **b** Box plot of percent methylation of CpG sites at *IPO13, COMMD6, SPB,* and *SLC2A4RG*. The y-axis for both box plots represents methylation level. Genes associated with the CpG are given in parentheses. Box plot represents the range of variation and median value. **c** Relative transcript amount that adjusts the transcript amount of Duroc to 100% as represented on the x-axis and compared with other breeds. The y-axis shows gene names. * *P* < 0.05, ** *P* < 0.01

In all 4 groups of pigs we analysed the level of expression in 8 genes, which were differentially methylated in at least 1 comparison: *SLC22A18*, *EHD3*, *TEDC2, NUDT7, IPO13, COMMD6, SPTB*, and *SLC2A4RG;* four of which showed differential expression between Duroc and PiNN in our previously microarray study (*IPOI3, COMMD6, NUDI7, SLC2A4RG*). All correspondent CpGs were located within 2 kb upstream the TSS except for *NUDT7* (distance of 5.6 kb; Additional file 9). Box plots of percent methylation of CpG sites on these genes are shown in Fig. 7a and b. Figure 7c shows the corresponding levels of expression as revealed by qPCR (*n* = 8–10 animals per breed). We obtained directionally consistent and significant correlations of 0.6 to 0.8 between the expression levels of microarrays of our previous study and qPCR with the same animals [18]. Most genes were significantly differentially expressed in one of the groups, except *SPTB* and *SLC2A4RG* (Fig. 7c). Three genes were differentially expressed among Pietrain breeds, including *SLC22A18*, *EHD3,* and *IPO13*. Significantly different expression was found between Duroc and Pietrain breeds for *TEDC2, NUDT7, IPO13,* and *COMMD6*. Correspondingly, CpG sites within these genes were also differentially methylated. In particular, high expression levels of *EHD3* with low levels of methylation and low expression levels of *NUDT7* with high levels of methylation were found in PiPP, with vice versa results in PiNN.

**Fig. 8:**
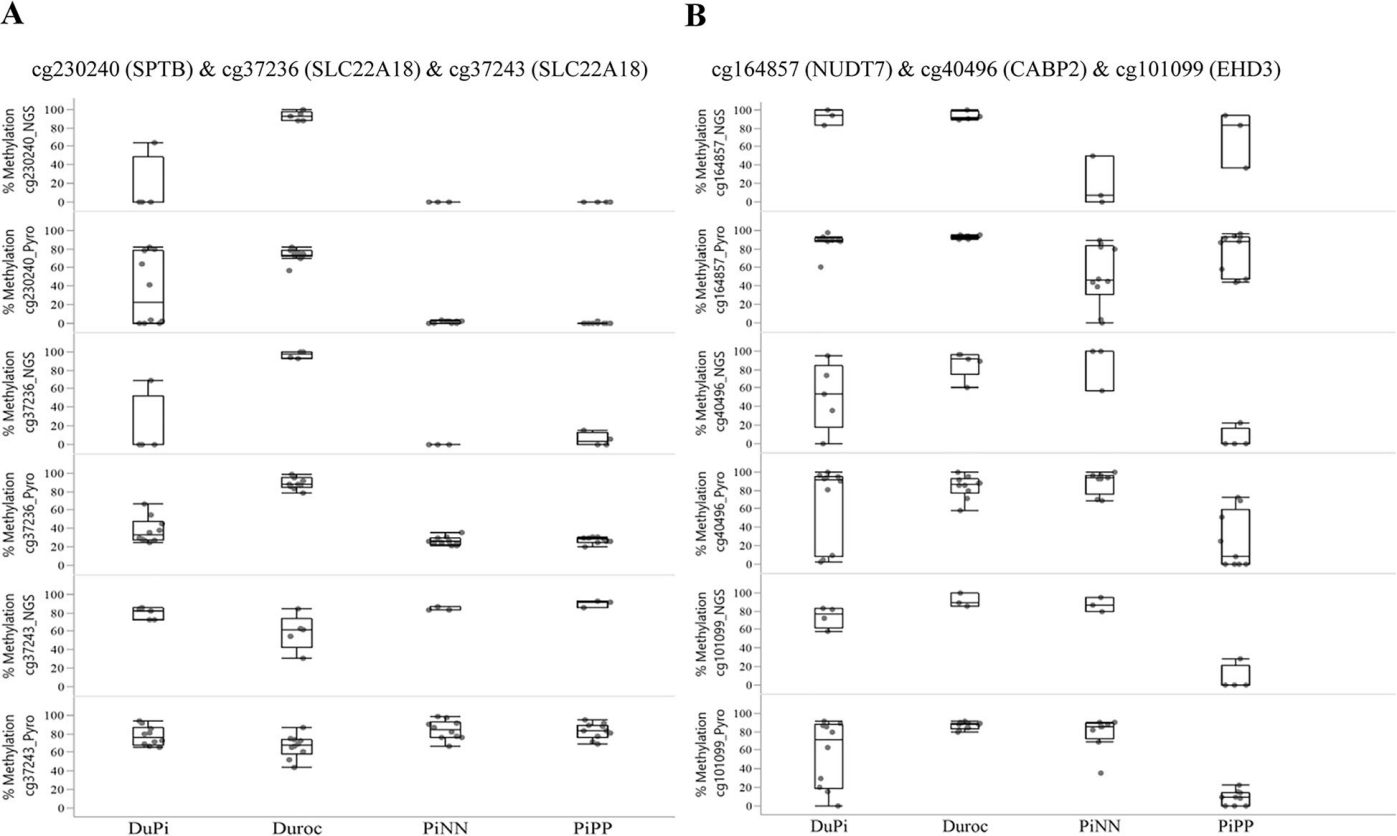
Comparison of differentially methylated CpG sites between bisulfite sequences from next generation sequencing data (NGS) and pyrosequencing data (Pyro). **a** Box plot showing percent methylation of CpGs site at *SPTB* and *SLC22A18*. **b** Box plot showing percent methylation of CpG sites at *NUDT7, CABP2,* and *EHD3*. The y-axis of both box plots represents degree of methylation. Genes associated with the CpG are given in brackets. Box diagram represents the range of variation and mean value. Each point represents a CpG of the individual used. The number of individuals used per breed was 3–5 for NGS and 8–10 for Pyro

Moreover, we used pyrosequencing with more samples (8–10 samples per breeds) to validate NGS data. Box plots compared pyrosequencing and NGS data of the CpGs sites at *SPTB* and SLC22A18 (Fig. 8a) and for *NUDT7, CABP2*, and *EHD3* (Fig. 8b). The Spearman correlation between NGS data and pyrosequencing at *p < 0.05* ranged 0.61–0.81 (*EHD3* r = 0.61, *p = 0.021*; *SPTB* r = 0.79, *p < 0.001*; *CABP2* r = 0.81, *p < 0.001*; *NUDT7* r = 0.63, *p = 0.019*; and cg37243 of *SLC22A18* r = 0.75, *p = 0.001*). Together, our data suggest good concordance between NGS data and pyrosequencing results.

## Discussion

Population-specific methylation has been demonstrated in humans, leading to an understanding of population-specific disease phenotypes [28]. Methylation-specific patterns in pig populations may have been promoted by selection for particular traits. Long-term selection and intensive breeding programs have led to a divergence of phenotypes in pigs, including leanness and fat content. Duroc and Pietrain pig breeds have undergone long-term selection to differentially favour traits related to meat and carcass quality, leading to substantial differences in leanness, muscularity, and fat content to represent divergent metabotypes. This study also included F2 crosses of the two divergent breeds, providing a first clue on those differential methylated sites that may have evolved due to the selection process and might be expected to show balanced methylation levels in crosses. The methylation sites that appear as either being of Duroc- or Pietrain-origin that were found based on the comparisons of DuPi vs. both pure breeds potentially represent sites contributing to breed differences since these methylation sites were transferred in crossbreeding over generations. These sites are at least loci that distinguish the pure lines analysed here, which are only exemplary for the breeds Duroc and Pietrain. The groups of PiPP and PiNN that we studied differ at the *RYR1* g.1843C > T genotype on SSC6. In Pietrain pigs (PiPP), mutations in ryanodine receptor 1 (*RYR1*) are associated with susceptibility to malignant hyperthermia syndrome (MHS) and reduced meat quality (pale, soft, exudative) [29, 30]. Mutation of the Ca 2+ release channel, encoded by RYR1, exhibited phenotypic consequences in muscle tissue and the whole organism mediated by modulated Ca^2+^ metabolism. The RYR1 mutation in pigs causes a dysregulation of the calcium-flux leading to early energy depletion, AMPK activation, accelerated glycolysis and an increased incidence of pale, soft, exudative (PSE) meat [31]. We believe that numerous mechanisms and processes likely compensate for the impact of the mutation on intracellular Ca^2+^ homeostasis. Therefore, epigenetic modifications of genes related to Ca^2+^ metabolism may be involved.

In our study, we showed differences in methylation patterns between populations that differ in metabolic phenotype and genetic background at a major gene. All 441,894 CpGs identified by our study can assemble the four groups in a hierarchical cluster analysis, confirming specific DNA methylation patterns of the groups. A potential bias of different DNA methylation among breeds may occur through the presence of SNPs. In this case, we deleted SNPs detected in DNA pools of the respective animal groups. Epigenetic variation detected by bisulfide sequencing may be confounded with genetic variation. For clearer results animals should be genotyped or better whole genome sequenced. In our study, we only sequenced a pool of animals of each breed that may have not recognize all SNPs. The highest number of different DNA methylation sites was found between Duroc and Pietrain pigs, in line with their highly different phenotypes. Methylation profiles of DuPi, the F2 crosses of the two pure breeds, showed intermediate differences. Comparisons between the two Pietrain groups, which differed in the *RYR1* locus only, revealed the lowest differences in DNA methylation patterns. This shows the relationship between DNA methylation pattern differences and phenotypic differences based on an infinite number of quantitative trait loci (QTL) on the one hand and based on a single major gene on the other hand. Previous studies have shown significant genetic control of transgenerational similarity in DNA methylation [32]. This exciting perspective informs our understanding of the link between genetics and the environment, which are in turn linked to phenotype.

The regions differentially methylated among many breeds involved CpGs at 63.38–63.39 Mb on SSC9. This region also showed strong heterogeneity in methylation and very pronounced change in methylation levels among breeds. The region (9:63272406–63,401,079 bp) contained large CpG islands (CGI) and contained many predicted TSS (genome assembly: Sscrofa11.1). TSS was defined using TSS Eponine track from Seqmonk [33]. According to SeqMonk at this position (9:63272406–63,401,079) the ratio of observed to expected CpG of CpG islands is 1.21. This long CGI may contain many other CGI clusters that co-localize more specifically to alternative TSSs and methylation domains [34]. Abnormal methylation of CGIs plays an important role in the regulation of gene expression as observed in many cancer types and regulation of tissue-specific genes [35–37]. Differential methylation in this CGI among many pig breeds may be due to breeding and selection.

Two interesting groups of different methylation patterns were observed based on metabolic phenotype (fatness and leanness) along with a distinct difference between Pietrain pigs with functional mutations in the skeletal muscle Ca^2+^ release channel *RYR* receptor. We found that differentially methylated genes between Duroc vs. other group were significantly enriched in TR/RXR activation. Thyroid hormone (T3) acts through the thyroid receptor (TR), forms heterodimers with RXR along with a number of co-activators, and affects a range of biological processes such as growth, development and metabolism. Moreover, perturbation of T3 and its receptors affects various processes including lipid metabolism, carbohydrate metabolism and steroid metabolism [34, 35]. Differentially methylated genes involved in the Wnt/Ca + pathway or calcium ion binding were found in Pietrain vs. other groups in particular PiPP vs. PiNN. This suggests that DNA methylation changes may induce functionally relevant changes in the skeletal muscle. Metrnl is a novel secreted protein and adipokine expressed in various tissues, including nervous system, adipose, muscle, and mucosal tissue. Metrnl also plays a role in lipid metabolism and insulin sensitivity [38]. In addition, Rao et al. reported a role for Metrnl as a circulating factor that is induced in muscle after exercise and in adipose tissue upon cold exposure, suggesting that Metrnl mediates muscle-fat crosstalk and immune-adipose interactions to increase beige fat thermogenesis [39]. In our study, the Duroc breed, which is fattier than Pietrain, had hypomethylation at cg300556, which maps to the 5′ region of *METRNL*. Hypo-methylation at cg300556 in Duroc pigs could thus be involved in increased expression of *METRNL* and could lead to high fat mass. This is in line with a previous study that found altered DNA methylation as a result of changes in lipid metabolism due to adiposity [40].

It was previously reported that porcine *IDH3B* is upregulated in the back fat of western commercial pigs compared to Chinese indigenous obese breeds and that a mutation in the promoter region induces increased porcine *IDH3B* expression [41]. In this study, two CpG sites (cg408473 and cg408474) located on the promoter region of *IDH3B* (− 14 and − 16 bp from TSS) were more methylated in Duroc and less methylated in the leaner Pietrain breed. The transcription factor CREB or AP-1 can bind in this position as revealed by LASAGNA-Search 2.0 of transcription factor binding sites (TFBSs) [42]. Our data suggest that DNA methylation variations at these CpG regions could potentially be responsible for adiposity. We also detected other genes, such as *COMMD6*, which show high expression levels and lower levels of CpG methylation in the promoter region (77 and 88 bp of TSS) in Duroc pigs. The COMMD family, including COMMD6, was recently described as novel regulatory molecules in plasma lipid metabolism [43]. Spectrin beta, erythrocytic (SPTB) plays a role in the stability of erythrocyte membranes and is associated with spherocytosis type 2, hereditary elliptocytosis, and neonatal hemolytic anemia [44]. *SPTB* was also reported as a sex-specific locus in an associated study of areal bone mineral density [45]. The CpG (cg230240) located in *SPTB* was unmethylated in both Pietrain breeds, highly methylated in Duroc, and hemimethylated in DuPi. However, the functional significance of this gene among pig populations remains unknown.

Imprinted genes are susceptible loci for environmentally induced diseases because of their functionally haploid nature [46]. This epigenetic mechanism leads to parent-of-origin silencing of alleles and depends mostly on DNA methylation and chromatin composition [47, 48]. Epigenetic differences among populations were also reported due to different methylation levels of imprinted genes, including *Igf2, H19,* and *MEG3* [49]. *SLC22A18*, an organic cation transporter, is paternally imprinted in humans and mice [50, 51]. Altered methylation patterns of several imprinted genes including *SLC22A18* lead to development of cancer or modified tumours [52]. Alcohol exposure during pregnancy also alters methylation patterns of *SLC22A18* [53]. Further, a link between *Slc22a18* and fat accumulation has been reported in rats [54]. In pigs, there is still limited knowledge about *SLC22A18*. Interestingly, *SLC22A18* located on QTL regions is associated with fat deposition and with lifetime reproductive traits [55]. Our study revealed higher methylation levels in Duroc compared to both Pietrain. However, only in PiPP expression and methylation levels showed a negative relationship. Many studies reported different methylation sites associated with expression, regardless of the directional change in expression and methylation level [27]. This may be due to the fact that DNA methylation is not exclusively associated with repression of transcription initiation [56]. Selection may play a significant role in altering methylation patterns in the imprinted gene *SLC22A18,* which may lead to phenotypic changes like fatness and altered reproductive traits. The differential methylation of imprinting is therefore likely based not only on environment but also results from breeding selection.

As described above that the differences between PiPP and PiNN are due to mutations within the *RYR1* selection. Gain-of-function mutations in *RYR* cause malignant hyperthermia. A recent study identified loss-of-function mutations in Ca^2+^-binding protein 2 (Cabp2) that causes recessive hearing loss [57]. CaBPs might also contribute to buffering free cytosolic Ca^2+^ ions and the lack of Cabp2-enhanced inactivation of Ca^2+^ influx in inner hair cells [57]. We found that cg40496 located on promoter regions of *CABP2* (− 77 bp from TSS) was more methylated in PiNN compared to PiPP. PiPP with defect in Ca2+ release channel ryanodine receptor (RYR) created to new molecular environment within the cell, which may lead to demethylated Cabp2 and contribute by buffering free cytosoloic Ca^2+^ ions in the cell. This gain-of-function mutation of *RYR* may play a role in the methylation profile of other related functional genes.

A recent study using causal analysis of genetic association supports changes in DNA methylation as a consequence and not cause of obesity [40]. The other transcript identified in our study with lower methylation in PiPP and higher methylation in PiNN that involves Ca^2+^ channel function was *EHD3*. EHD proteins are expressed in cardiac muscle and play key roles in membrane protein targeting and regulation [58]. *EHD3* is a key regulator of anterograde trafficking of the Na^+^/Ca^2+^ exchanger, targeting voltage-gated L-type Ca^2+^ channels in the cardiac ventricle and voltage-gated T-type Ca^2+^ channels in the atria [59, 60]. Together, causal SNPs in *RYR* may affect other related functional genes.

The other interesting transcript identified by our study was *NUDT7*, a member of the nudix hydrolase family. The difference in meat colour between Japanese wild boar and Large White pig breed was reported to be caused by partially different expression of this candidate gene located in the meat colour QTL region of SSC6 [61, 62]. In this study, *NUDT7* was also differentially expressed between Duroc and Pietrain as well as between Pietrain breeds. In addition, methylation levels of cg164857 located within 5671 bp of the TSS on *NUDT7* was also differentially methylated between breeds. *KIF3C* is a member of the *KIF3* family and functions as a motor protein involved in axonal transport in neuronal cells and myogenesis in muscle cells. *KIF3C* is expressed in proliferating myotubes of C2C12 cells, a rat myogenic cell line, as well as in adult mouse muscle [63, 64]. *KIF3C* was identified as an injury-specific kinesin that contributes to axon growth and regeneration by regulating organization of the microtubule cytoskeleton [65]. In our study, cg102313 located 1837 bp from TSS in *KIF3C* was highly methylated in PiPP but not in PiNN. How kinesin-II works together with RYRs is still unknown.

## Conclusions

DNA methylation variations could be mediated by metabolic type, as shown by the change in methylation profile of CpGs located in the promoter regions of *METRNL, IDH3B, COMMD6,* and *SLC22A18,* which are involved in lipid metabolism. In Pietrain pigs with functional mutations in the skeletal muscle Ca^2+^ release channel *RYR*, methylation of related functional genes like *CABP2* and *EHD* are affected*.* These genes may in turn be involved in buffering free cytosolic Ca^2+^ ions or trafficking of the Na^+^/Ca^2+^ exchanger. This study highlights DNA methylation differences among populations that may be the result of the selection process or a consequence of major gene mutation which play significant role in muscle phenotypes.

## Additional files

Additional file 1: Phenotype of the animals used in the study. (XLSX 14 kb)

Additional file 2: Primers sequence for pyrosequence and qPCR (XLSX 14 kb)

Additional file 3: CpG sites significant at FDR < 5% and different in methylation level > 25% between Dupi and Duroc (XLSX 108 kb)

Additional file 4: CpG sites significant at FDR < 5% and different in methylation level > 25% between Dupi and PiPP (XLSX 111 kb)

Additional file 5: CpG sites significant at FDR < 5% and different in methylation level > 25% between Dupi and PiNN (XLSX 123 kb)

Additional file 6: CpG sites significant at FDR < 5% and different in methylation level > 25% between Duroc and PiNN (XLSX 180 kb)

Additional file 7: CpG sites significant at FDR < 5% and different in methylation level > 25% between Duroc and PiPP (XLSX 179 kb)

Additional file 8: CpG sites significant at FDR < 5% and different in methylation level > 25% between PiNN and PiPP (XLSX 89 kb)

Additional file 9: CpGs differently methylated between Duroc and PiNN and the transcripts close to these CpGs (XLSX 27 kb)

Additional file 10: Genes annotated from the selected CpG (different methylation level > 25% and significant at FDR < 5% and position < 2 kb from TSS) were included in GO enrichment analysis by using online tool DAVID. (XLSX 16 kb)

## Data Availability

Expression data are available in the Gene Expression Omnibus public repository with the GEO accession number GSE69840: GSM1709900–GSM1709919. All RRBS sequencing data have been deposited in the ArrayExpress database at EMBL-EBI (www.ebi.ac.uk/arrayexpress) under the accession number E-MTAB-7338.

## Abbreviations

Dupi: F2 generation of cross-breeding between Duroc and Pietrain breeds
FDR: False discovery rate
MHS: Malignant hyperthermia syndrome
PiNN: Pietrain pig carries genotype 1843 C/C of *RYR1*
PiPP: Pietrain pig carries genotype 1843 T/T of *RYR1*
RRBS: reduced-representation bisulfite sequencing
RYR: Ca2+ release channel ryanodine receptor
TSS: Transcription start site
